# A Rare Case of Adenocarcinoma of the Urethra

**DOI:** 10.7759/cureus.87610

**Published:** 2025-07-09

**Authors:** Muhammad Waqas Shafiq, Taha Ahmad, Rabbya Naseem, Aisha Asif, Khurram Mir

**Affiliations:** 1 Surgical Oncology, Shaukat Khanum Memorial Cancer Hospital and Research Centre, Lahore, PAK; 2 General Surgery, Shaukat Khanum Memorial Cancer Hospital and Research Centre, Lahore, PAK; 3 General Practice, Dow University of Health Sciences, Lahore, PAK

**Keywords:** adenocarcinoma, chemotherapy, primary urethral cancer, rare urologic tumor, transurethral resection

## Abstract

Primary urethral adenocarcinoma is a rare and aggressive subtype of primary urethral carcinoma. We report the case of a 37-year-old man with a three-month history of painless hematuria. An initial superficial biopsy revealed poorly differentiated carcinoma with squamous features, but further histopathology after transurethral resection confirmed high-grade anterior urethral adenocarcinoma (T1 N0 Mx) without regional or distant metastasis. After multidisciplinary discussion, urethrectomy with perineal urethrostomy was recommended; however, the patient declined surgery and chose systemic cisplatin-gemcitabine chemotherapy instead.

## Introduction

In the United States, the annual age-adjusted incidence of primary urethral carcinoma (PUC) is estimated to range from 1.5 to 4.2 cases per million individuals [1]. According to the current World Health Organization classification, PUC is categorized into three principal histological subtypes, namely, urothelial carcinoma (55%), squamous cell carcinoma (21.5%), and adenocarcinoma (16.4%) [2]. Urethral adenocarcinoma represents the rarest and most aggressive form, originating from the urethral glands or their ducts [3]. In men, urethral adenocarcinoma frequently presents with nonspecific symptoms, such as hematuria, dysuria, or urinary obstruction, which often contributes to delayed diagnosis [4]. Due to its rarity and insidious presentation, the disease is commonly detected at an advanced stage [5]. Management typically involves surgical resection, radiation therapy, and chemotherapy; however, the overall prognosis remains poor owing to the high risk of local recurrence and distant metastasis [6]. Here, we present a case of primary urethral adenocarcinoma currently being managed with chemotherapy.

## Case presentation

A 37-year-old male shopkeeper presented with a three-month history of painless hematuria. The patient reported no weight loss, back pain, or changes in appetite, and his urinary stream remained normal. He was a nonsmoker with no history of Neswar use. He was married with six children and had no family history of cancer. His past medical history included a benign thyroidectomy in 2012. He had no history of hypertension or diabetes mellitus and was not on any regular medications.

**Figure 1:**
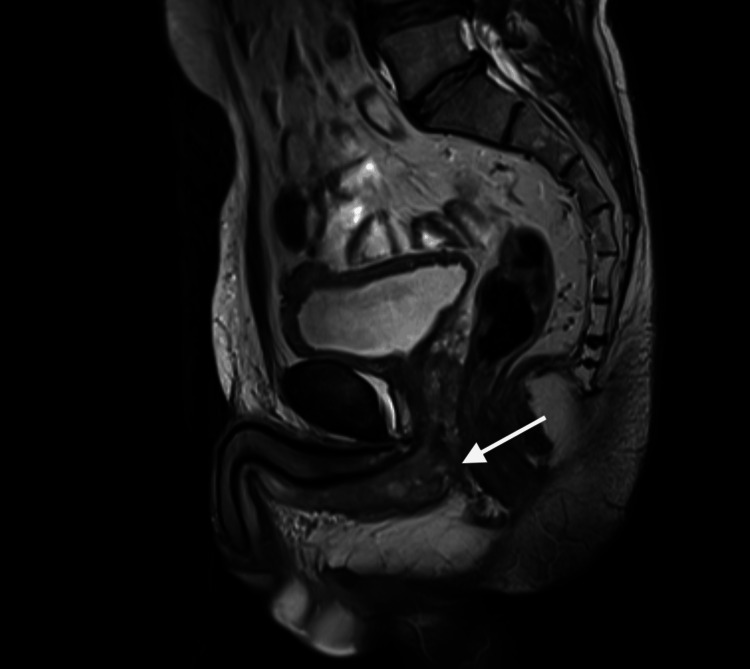
Urethral mass on MRI saggital view (white arrow).

**Figure 2:**
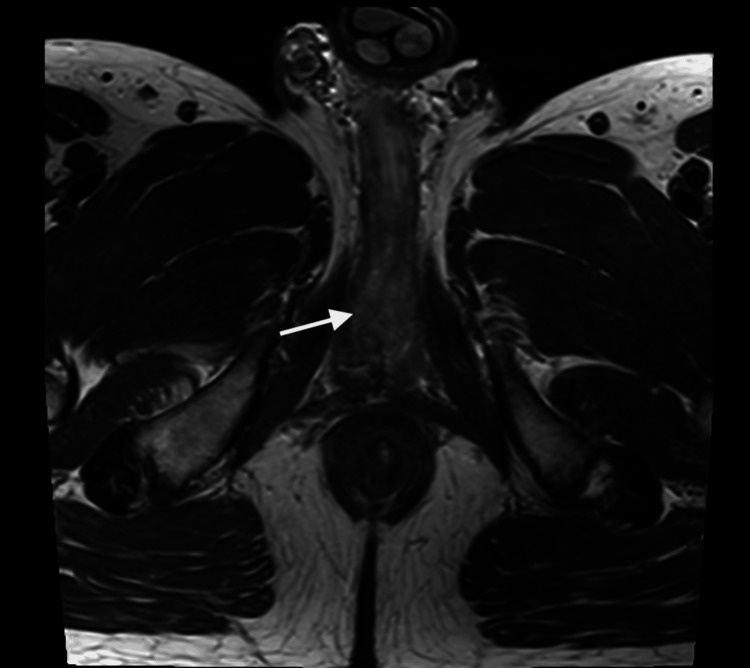
Urethral mass on MRI axial view (white arrow).

The patient underwent a cystoscopy and biopsy of the urethral lesion at an outside facility, which showed a scanty superficial sample with poorly differentiated carcinoma exhibiting squamous differentiation. Given the limited tissue sample, a larger biopsy was recommended for a definitive diagnosis. Subsequently, an MRI of the pelvis revealed biopsy-proven anterior urethral carcinoma, without invasion of the periurethral muscles, prostate, or urinary bladder (Figures 1, 2). No enlarged lymph nodes were observed, and the tumor was staged as T1 N0 Mx. A CT scan of the chest and abdomen confirmed there were no signs of distant visceral or osseous metastasis.

**Figure 3:**
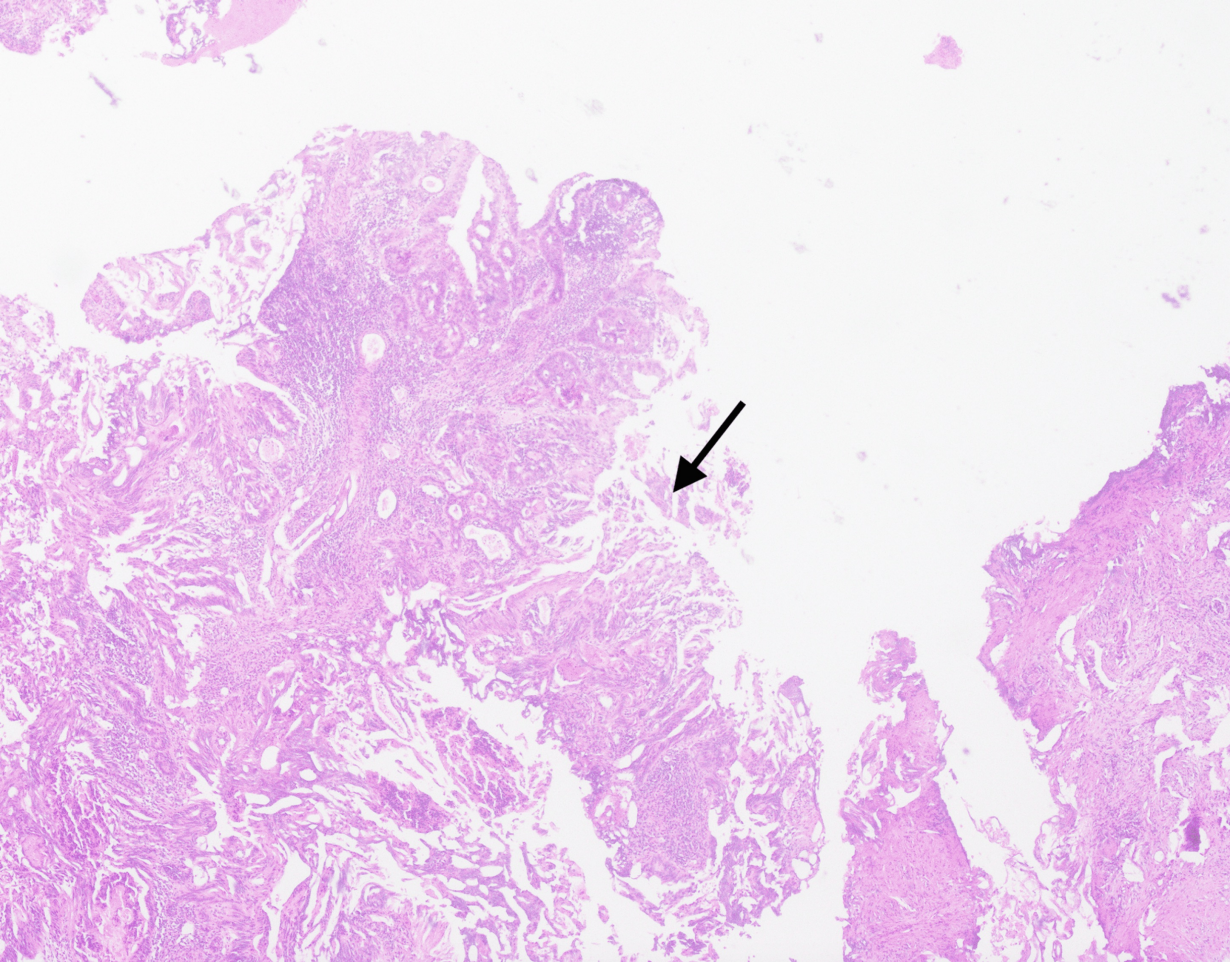
Histopathology (black arrow).

The patient was admitted for cystoscopy and underwent a transurethral resection of the urethral tumor. During the procedure, a tight meatus was noted, with a single lesion measuring approximately 1.5 cm at the bulbo-membranous junction, partially occluding the urethral lumen. The high bladder neck was observed, but no tumor was present in the bladder, and bilateral ureteric orifices were visualized. Histopathological examination of the tumor biopsy taken revealed high-grade adenocarcinoma (Figure 3).

The case was discussed in a multidisciplinary team (MDT) meeting, where recommendations included obtaining an MRI of the liver, prostate-specific antigen (PSA) levels, and a digital rectal examination (DRE). However, the DRE was not performed due to a fissure at the 6 o'clock position. An MRI of the abdomen revealed segment VIII hemangiomas, with no evidence of hepatosplenic metastatic deposits. PSA level was 0.33 mg. Following further MDT discussions, the patient was advised to consider urethrectomy and perineal urethrostomy formation, with the alternative option being referral to medical oncology for chemotherapy if he declined surgery.

After a detailed discussion outlining the potential benefits and risks of surgical management versus systemic therapy, the patient declined urethrectomy and perineal urethrostomy and instead opted for systemic chemotherapy. He is currently planned to receive combination chemotherapy with cisplatin and gemcitabine. This case highlights the diagnostic challenges and the importance of individualized management in this rare malignancy.

## Discussion

Primary urethral adenocarcinoma is not only rare but also poorly characterized due to the paucity of robust population-based studies and prospective trials [7]. Due to its rarity and nonspecific presentation, delays in diagnosis are frequent and may adversely affect prognosis.

As Dalbagni et al. [7] and Rabbani [8] describe, distinguishing primary urethral adenocarcinoma from secondary involvement by prostatic or colorectal adenocarcinoma can be challenging, particularly in men. Immunohistochemical staining panels, such as PSA, CK7, CK20, and CDX2, may be valuable adjuncts in confirming urethral origin and excluding mimickers such as colorectal adenocarcinoma [9]. Basiri et al. [10] and Gatta et al. [11] also highlight that inadequate or superficial biopsy samples may misrepresent the tumor’s true histology, emphasizing the need for sufficient deep tissue sampling, as was ultimately performed in this case.

While radical surgery remains the preferred option for localized disease, the literature indicates that up to a third of patients may not undergo surgery due to advanced stage at presentation, comorbidities, or personal choice [7]. This patient’s decision to decline urethrectomy reflects one of the real-world challenges clinicians face when balancing oncologic control with patient preferences, quality of life, and psychological readiness for potentially mutilating procedures.

Chemotherapy regimens for primary urethral adenocarcinoma are largely extrapolated from protocols used for other urothelial or glandular malignancies. Cisplatin-based combinations, such as cisplatin plus gemcitabine, have been employed with variable outcomes in small series and case reports [10]. In this case, after comprehensive multidisciplinary counseling, the patient declined surgical intervention and opted for chemotherapy. Given the tumor’s aggressive nature and high risk of recurrence and metastasis, close follow-up with periodic imaging and cystoscopy is warranted to monitor treatment response and detect disease progression [12].

In summary, primary urethral adenocarcinoma represents a unique diagnostic and therapeutic challenge with limited consensus on optimal management. This case adds to the small but growing body of evidence demonstrating the need for early and adequate biopsy, thoughtful use of immunohistochemistry, shared decision-making, and careful follow-up. Further multicenter studies and pooled analyses are needed to refine treatment algorithms and improve outcomes for this rare and aggressive malignancy.

## Conclusions

Primary urethral adenocarcinoma is rare and diagnostically challenging, often requiring a high index of suspicion. This case highlights the importance of thorough biopsy sampling, appropriate immunohistochemistry, and multidisciplinary input to ensure accurate diagnosis and treatment planning. It also emphasizes that while surgery is the standard for localized disease, systemic chemotherapy may be considered when surgery is not feasible. By sharing this experience, we hope to increase clinical awareness and contribute to more informed diagnostic and management pathways for this uncommon malignancy.
